# ANTI-AGING PROTEIN CD9 AFFECTS AGE-RELATED HEART FAILURE

**DOI:** 10.1093/geroni/igz038.953

**Published:** 2019-11-08

**Authors:** Sriya T Jonnakuti, Mujib Ullah

**Affiliations:** Stanford University, Palo Alto, California, United States

## Abstract

The CD9 is transmembrane protein that plays a critical role in many cellular processes including aging associated cardiac pathologies. The heart function declines in the aged population. Ageing is strongly associated with many age-related conditions such as increased risk of heart failure. If aging can be prevented slowed down or even reversed, heart failure and other signs of aging could be controlled or even cured. It is unknown whether CD9 is cardioprotective. The objective of this study is to investigate whether a decline CD9 levels contributes to aging-related heart failure. Our data shows that CD9-deficient aged mice develop cardiac abnormalities and pathological cardiac hypertrophy, Cardioprotection by CD9 in old mice is followed by the downregulation of SIRT6 in the heart, and CD9 overexpressed exosomes ameliorates cardiac pathologies in treated mice and improves their long-term survival. Additionally, the serum level of CD9 decreased significantly in aged mice. CD9 overexpressed exosomes are cardioprotective and improve cardiac function in aged mice. These exosomes mediate their paracrine effects by attenuating, blood pressure, heart beat, reactive oxygen species and fibrosis. Remarkably, CD9 overexpression reversed fibrosis associated brain natriuretic peptide (BNP), Sirt6, and galectin 3 (Gal-3). These results provide a new perspective on the pathogenesis of cardiomyopathies and open new avenues for treatment of the disease.

